# Comparison of methodologies for modeling directional deep brain stimulation electrodes

**DOI:** 10.1371/journal.pone.0260162

**Published:** 2021-12-15

**Authors:** Anneke M. Frankemolle-Gilbert, Bryan Howell, Kelsey L. Bower, Peter H. Veltink, Tjitske Heida, Cameron C. McIntyre

**Affiliations:** 1 Department of Biomedical Engineering, Case Western Reserve University, Cleveland, OH, United States of America; 2 MIRA Institute for Biomedical Engineering and Technical Medicine, University of Twente, Enschede, The Netherlands; Vellore Institute of Technology: VIT University, INDIA

## Abstract

Deep brain stimulation (DBS) is an established clinical therapy, and directional DBS electrode designs are now commonly used in clinical practice. Directional DBS leads have the ability to increase the therapeutic window of stimulation, but they also increase the complexity of clinical programming. Therefore, computational models of DBS have become available in clinical software tools that are designed to assist in the identification of therapeutic settings. However, the details of how the DBS model is implemented can influence the predictions of the software. The goal of this study was to compare different methods for representing directional DBS electrodes within finite element volume conductor (VC) models. We evaluated 15 different DBS VC model variants and quantified how their differences influenced estimates on the spatial extent of axonal activation from DBS. Each DBS VC model included the same representation of the brain and head, but the details of the current source and electrode contact were different for each model variant. The more complex VC models explicitly represented the DBS electrode contacts, while the more simple VC models used boundary condition approximations. The more complex VC models required 2–3 times longer to mesh, build, and solve for the DBS voltage distribution than the more simple VC models. Differences in individual axonal activation thresholds across the VC model variants were substantial (-24% to +47%). However, when comparing total activation of an axon population, or estimates of an activation volume, the differences between model variants decreased (-7% to +8%). Nonetheless, the technical details of how the electrode contact and current source are represented in the DBS VC model can directly affect estimates of the voltage distribution and electric field in the brain tissue.

## 1. Introduction

Deep brain stimulation (DBS) is an established therapy for the treatment of movement disorders and shows promise for the treatment of neuropsychiatric disorders [1]. Clinical DBS technology is advancing with the introduction of new electrode designs [2]. Traditional DBS leads consisted of four cylindrical electrode contacts, whereas newer directional leads segment the cylindrical band into three separate electrode contacts. These segmented electrodes show promise in steering stimulation toward therapeutic areas and away from areas that are known to generate side effects [3]. As such, directional DBS electrodes can increase the therapeutic window of clinical stimulation by lowering the efficacy threshold and increasing the side effect threshold [4].

While the technical advantages of directional DBS leads are relatively straightforward, the clinical advantages are less obvious because the increased complexity of the lead makes it more difficult to search the parameter space and identify an optimal therapeutic setting. Therefore, computational models have been proposed as tools that could assist in clinical programming [5]. Patient-specific DBS models provide insight on the neural response to the applied voltage distribution and the modulation of different brain pathways by DBS [6]. These computational tools have also been used to guide the surgical targeting [7] and clinical programming of DBS therapy [8].

Computational models of DBS generally rely on finite element methods (FEM) to solve for the voltage distribution in the brain from the DBS pulses [9]. The model used to solve for the DBS voltage distribution is known as a volume conductor (VC) model. The voltage distribution results from the VC model can then be coupled to neuron models (typically axons) to quantify a simulated biophysical response to DBS [10]. The coupled VC-neuron simulation is known as a field-cable model. One drawback of DBS field-cable models is that they are relatively complicated and computationally demanding. Therefore, more simplified metrics like an electric field isosurface (e.g. 0.2 V/mm) [11], or a volume of tissue activated (VTA) calculation [12], have been proposed to provide a generalized estimate of stimulus spread. These simplified metrics are derived from the DBS VC voltage distribution data, and are popular in clinical DBS research studies [13], but carry with them a sizeable list of limitations [14].

Understanding the limitations and assumptions of a given DBS model becomes relevant when attempting to define correlations between the simulated neural response to DBS and actual behavioral effects measured in patients [13]. As such, DBS model development is often seeking a balance between biophysical realism and computational simplicity, but the functional optimum along that continuum is not obvious [10]. For example, previous studies have demonstrated that the inhomogeneity, anisotropy, and permittivity of the brain tissue medium can dramatically affect the simulated voltage distribution in DBS VC models [15]. However, implementing those kinds of electrical details in a clinical software tool are not realistic [14]. Therefore, the common assumptions in clinical analyses are to model the brain tissue as an isotropic medium and use electrostatic solutions of the DBS voltage distribution [16]. Nonetheless, the details of how the electrode contacts and current sources are represented in the finite element model can also affect the voltage distribution solution [17]. A direct comparison of implementation strategies for representing the DBS electrode contacts and current sources in DBS VC models is not currently available in the literature. Therefore, the goal of this study was to compare different finite element modeling methodologies to simulate current-controlled DBS from directional electrodes.

## 2. Methods

### 2.1 Volume conductor model

A finite element human head model, based on Howell and McIntyre [18], was used to compare the effects of using different modeling methodologies for simulating current controlled stimulation with a clinical directional DBS lead (Boston Scientific 2202). Fifteen different variants of the volume conductor (VC) model were defined, which included five different methods for representing the current source for the active contact, and up to four different options for representing the DBS electrode contact (section 2.2). The voltage distribution (*V*_*e*_) was calculated for each of the VC model variants by solving Laplace’s equation using the finite element method in COMSOL Multiphysics (v5.5).

For every VC model, the DBS lead was surrounded by a 0.5 mm encapsulation layer with an isotropic conductivity of 0.13 S/m. Each model variant also included a head domain and a brain domain which were defined from the Multimodal Image-based Detailed Anatomical (MIDA) model of the human head and neck [19]. The brain domain was defined as the area between the encapsulation layer and the outer boundary of the brain. The brain tissue was modeled as a homogeneous isotropic medium with a conductivity of 0.2 S/m. The head domain consisted of the area between the brain and the scalp and was modeled as ten heterogeneous structures based on MIDA_12_, where each structure had its own isotropic conductivity [18]. The outer boundary of the head was insulated except for the base of the neck, which was set to ground.

### 2.2 Directional DBS lead

**Fig 1:**
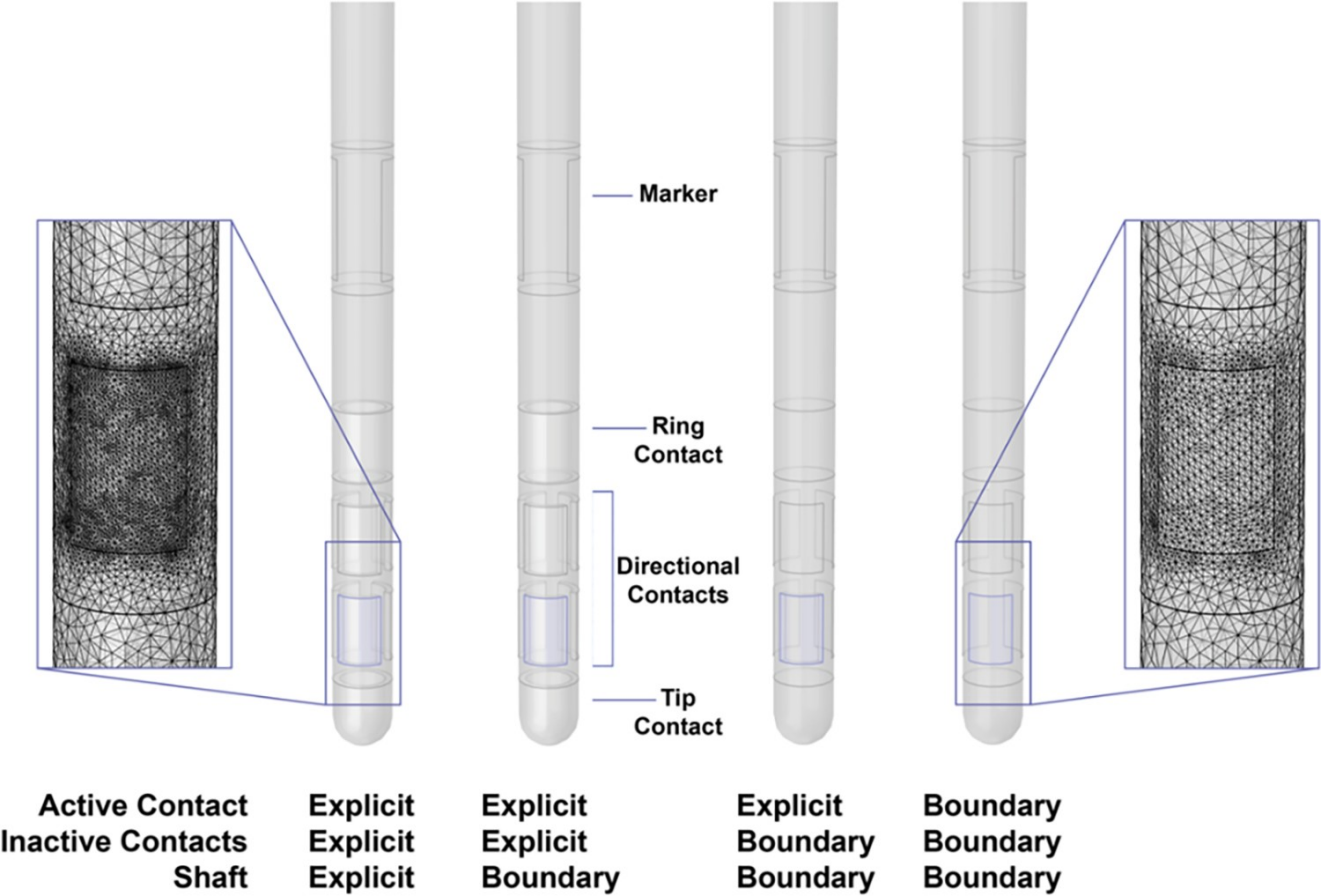
DBS lead design. The directional DBS lead was modeled in COMSOL with either an explicitly modeled or boundary representation for the active contact, inactive contacts, and shaft. The insets show mesh details for the explicitly modeled lead (left) and the lead represented by boundaries (right).

The directional DBS lead consisted of one cylindrical contact, six directional contacts, and one tip contact (Fig 1). Our simulations were performed with one active contact and seven inactive contacts. The geometry of each component of the DBS lead was either explicitly built or defined as a boundary in the model (Table 1 and Fig 1). Explicit components existed as a domain with assigned material properties. When modeled explicitly, the shaft was an insulator with a conductivity of 1e-16 S/m, and the contacts were modeled as platinum/iridium with a conductivity of 5.3e6 S/m. The boundary components were built and consequently subtracted from the DBS lead model which resulted in external boundaries that were defined using boundary conditions. The inactive contacts were treated as ideal conductors and modeled using Robin boundary conditions, which specified that all potentials within the contact were equal in value and that the net current flow through the surface of the contact was 0 mA.

**Table 1:** COMSOL models.

	Source	Geometry		
Model #	Active Contact	Active Contact	Inactive Contacts	Shaft
**1**	Point Current Source	Explicit	Explicit	Explicit
**2**	Point Current Source	Explicit	Explicit	Boundary
**3**	Point Current Source	Explicit	Boundary	Boundary
**4**	Boundary Current Source	Explicit	Explicit	Explicit
**5**	Boundary Current Source	Explicit	Explicit	Boundary
**6**	Boundary Current Source	Explicit	Boundary	Boundary
**7**	Current Density	Boundary	Boundary	Boundary
**8**	Electric Potential	Explicit	Explicit	Explicit
**9**	Electric Potential	Explicit	Explicit	Boundary
**10**	Electric Potential	Explicit	Boundary	Boundary
**11**	Electric Potential	Boundary	Boundary	Boundary
**12**	Floating Potential	Explicit	Explicit	Explicit
**13**	Floating Potential	Explicit	Explicit	Boundary
**14**	Floating Potential	Explicit	Boundary	Boundary
**15**	Floating Potential	Boundary	Boundary	Boundary

One of five current sources was used for the active contact. The DBS lead geometry was modeled with either an explicit or boundary representation for the active contact, inactive contacts, and shaft. Inactive contacts were always modeled using a floating potential.

To generate a default solution for each VC model, the active electrode contact was set as a cathode to deliver 1 mA current. The stimulus was defined as either a point current source, boundary current source, current density, electric potential, or floating potential. The point current source model was implemented by placing a point source in the center of the active contact domain. The boundary current source adds a source to the interior boundary of the active contact domain, which requires an explicit model of the active contact. The boundary current source was defined by a Neumann boundary condition, based on the known surface area of the active contact. The current density source model is only applicable to exterior boundaries, so this source requires a boundary representation of the active contact geometry. The current density source was defined by a Neumann boundary condition of the current density on the active contact surface, based on the known surface area of the active contact. The electric potential source model was implemented by solving the VC model twice. The model was first solved with Dirichlet boundary conditions of 1 V at the active contact, and the output current was calculated by integrating the current density over the surface area of the active contact. The model was then solved a second time with a 1 mA output current by setting the Dirichlet boundary condition as the reciprocal of the output current. The floating potential source was implemented as an equipotential boundary by assigning a Robin boundary condition set to 1 mA. The number of options for modeling the geometry of the DBS lead as either an explicitly modeled domain, represented as a boundary condition, or a combination of both, depended on which method was used to represent the current source. Table 1 lists the model variants used in this study.

**Fig 2:**
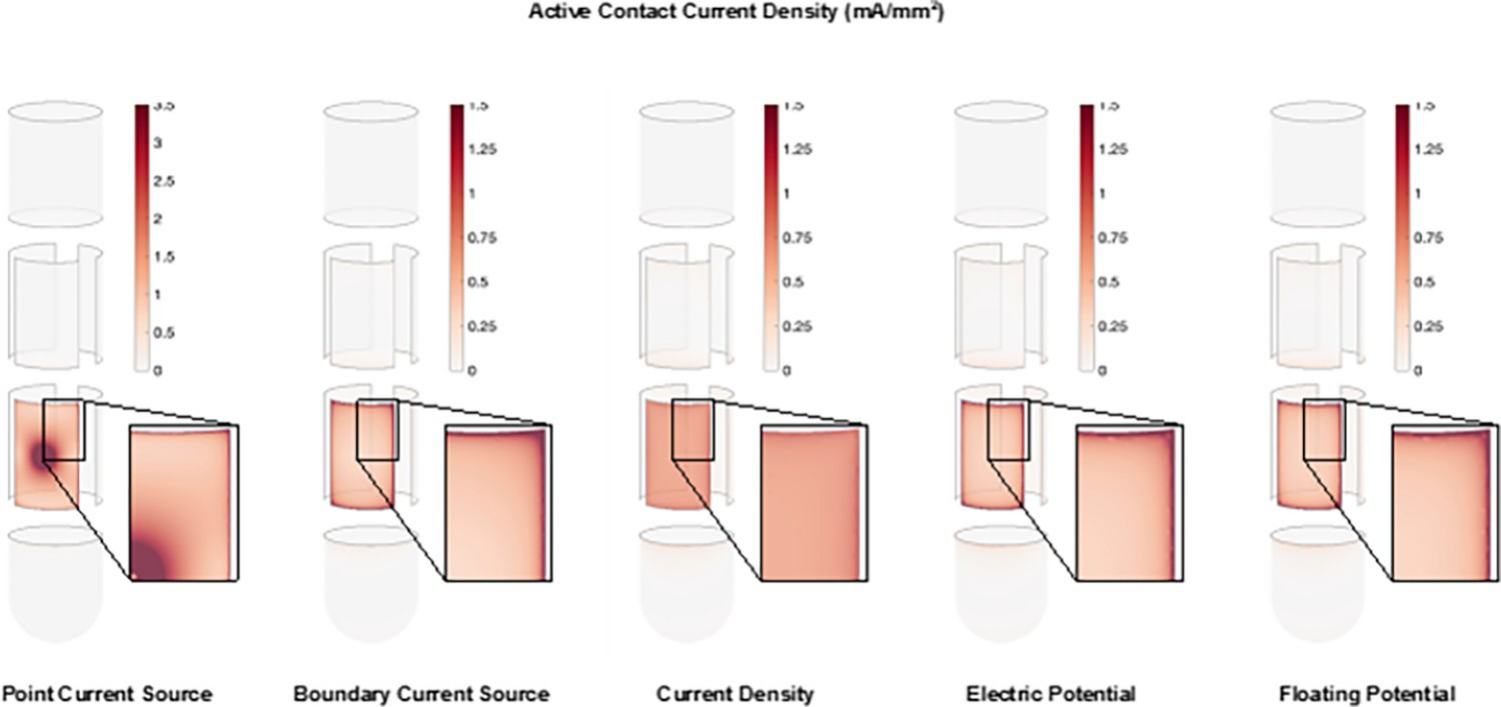
Active contact current density. The current density on the active contact for each of the current sources. The inset shows a closer view of the corner area of the contact.

The different VC models were compared with an output current of 1 mA on the active contact. The active contact current was calculated by integrating the normal component of the current density over the active contact surface (Fig 2). The formulas used for each current source are included as S1 Table. It should be noted that COMSOL applies the various current source models differently (S1 Table). For example, the boundary current source adds a discontinuity across the boundaries so both sides of the boundary have to be evaluated to determine the current on the contact surface. COMSOL defaults to taking the average instead of the difference when calculating the surface current, so it is important to consider how the initial current source is applied.

Since the current through the active contact is integrated over the contact surface it is important that the mesh is sufficiently fine to ascertain the active contact output current equals the input current. Additionally, the mesh on the active contact surface has to be the same for each model variant to accurately compare the different models with each other. To accomplish this across the VC model variants, the active electrode surface was always meshed first to generate our desired mesh, then we would mesh the remaining components of the DBS lead. A tetrahedral mesh was used in all domains and the resolution of the mesh was chosen so that increasing the resolution resulted in a change of less than 21% in the calculated voltage distribution.

The insets in Fig 1 show a mesh detail for both the explicitly modeled DBS lead (left) and the DBS lead modeled using boundary conditions (right). The more complex models explicitly modeled the contacts and the shaft, so they had an increased mesh density overall. However, the mesh on the surface of the contact was the same in all models. Model # 1 was considered to be the closest to physical realism and it was therefore used as the reference case for this study (Table 1).

### 2.3 Axon models

**Fig 3:**
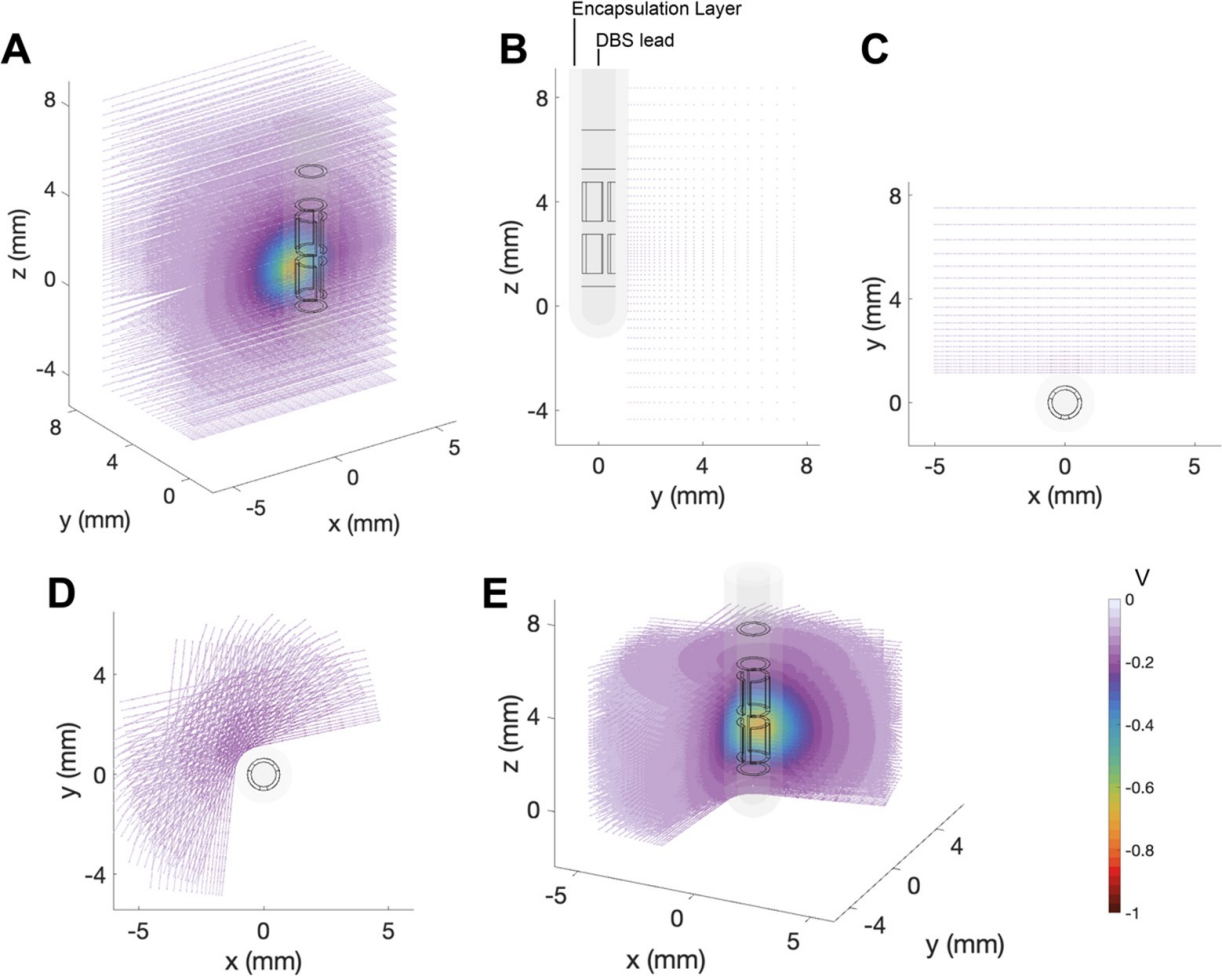
Axon models. Representation of the axons with respect to the DBS lead which is paced in an encapsulation layer. The potential distribution is imposed on the axons. **A**. 3D view of the axon grid perpendicular to the DBS lead and centered around the active directional contact. The YZ view (**B**) and XY view (**C**) of the DBS lead, encapsulation layer and axon grid. A subset of the axon grid is rotated around the DBS lead. The XY view (**D**) and 3D view (**E**) show the DBS lead, encapsulation layer and the rotated axon grid with the potential distribution imposed on the axons.

Axon models were used to estimate a neural response to the DBS voltage distribution (Fig 3). A large population of straight axon models were orientated perpendicular to the DBS lead. The grid of axons was centered on the active contact and distributed logarithmically with increasing distance from the electrode (Fig 3B and 3C). A subset of the grid was rotated around the DBS lead to capture the spatial effects of stimulation through the directional electrode contacts (Fig 3D and 3E). The potential distribution from a cathodic 1 mA DBS pulse was solved in the VC model and interpolated along each axon model as static extracellular potentials (Fig 3A). The static extracellular potentials were scaled by the time-varying stimulus waveform to create a vector describing the extracellular potential along the axon over time for the given stimulus pulse. A monophasic rectangular stimulus waveform of 60 μs was used in this study, and the stimulus amplitude of the pulse was adjusted to identify the threshold for activation of each axon model.

Each axon was simulated as a multi-compartment cable model of a myelinated axon [20], which was defined in NEURON (v7.3) [21]. The cable model enabled calculation of the membrane response to a given DBS stimulus. An axon model was deemed activated by a given stimulus amplitude if the DBS pulse, which was derived from the VC model, generated an action potential that propagated through the axon. Stimulation amplitudes were iteratively evaluated until the activation threshold was identified for each individual axon in the population.

## 3. Results

There are many different ways to construct VC models for DBS leads. This study evaluated four different contact geometry designs (Fig 1), with five different source implementations (Fig 2). Each source implementation produced a different current density distribution on the active contact. The current density was always higher at the edges and corners of the contacts than in the middle of the contact, except when using a point current source (Fig 2). The point current source exhibited a high current density at the center of the electrode contact, where the point source was located, as well as a high current density at the edges and corners of the contact.

**Fig 4:**
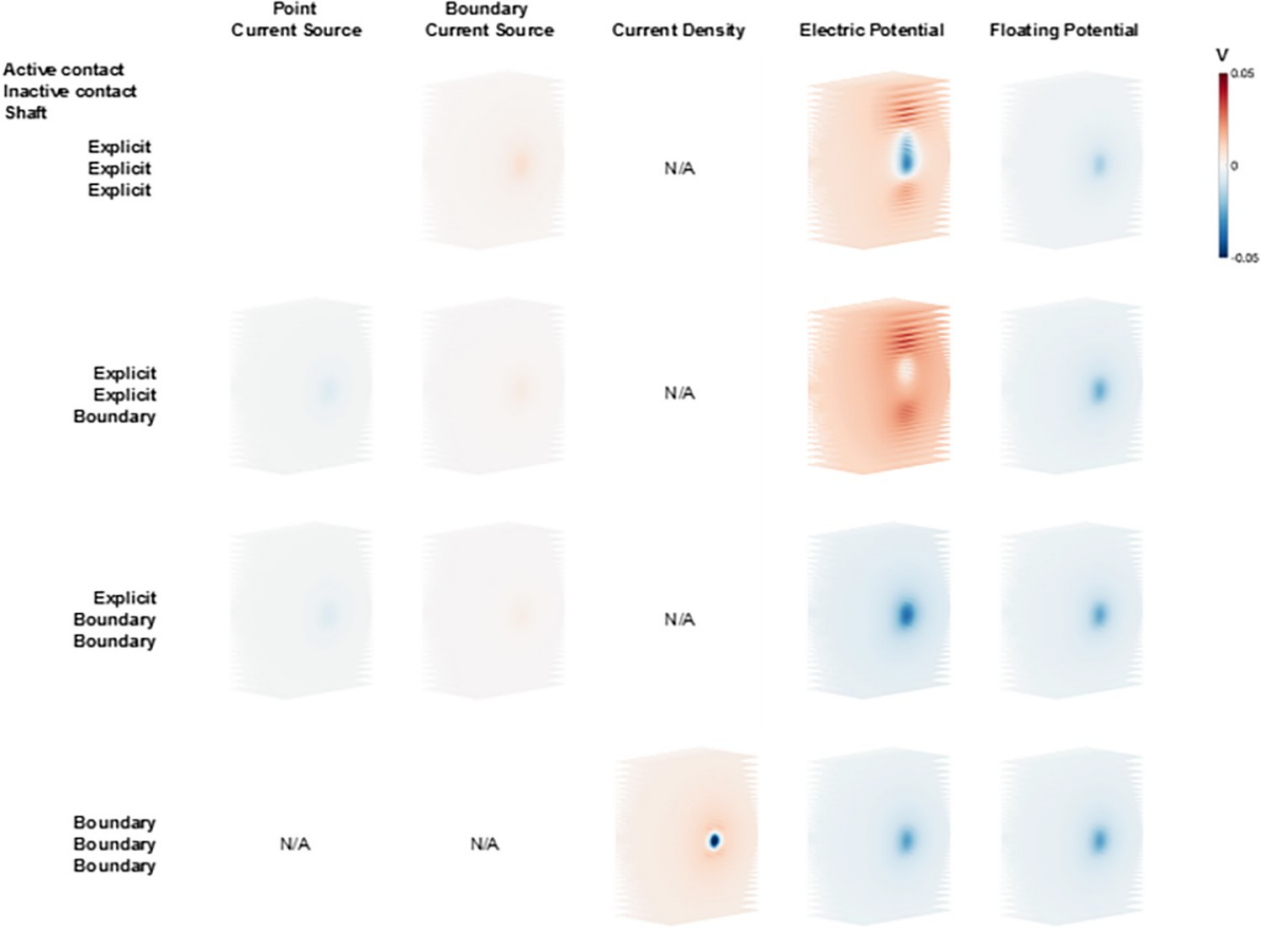
Voltage distribution differences. Voltage distribution of all model variants relative to Model #1 imposed on a perpendicular axon grid (Fig 3A). All models were solved with the same directional contact set as the active contact at 1 mA. The columns show the different current sources, and the rows show the different geometry presentations of the DBS lead. N/A indicates an inapplicable combination of source and geometry representation.

Given that the basic purpose of DBS is to modulate neural activity, we used axonal activation as an output metric to compare the model variants. Axonal activation is dictated by the DBS voltage distribution generated along the trajectory of the axon. Therefore, the first step in our analysis was to compare the DBS voltage distribution applied to a grid of axons positioned near the DBS lead (Fig 3). Fig 4 shows the differences in voltage distribution of each DBS electrode model variant applied to one example grid of axons that were orientated perpendicular to the active directional contact. These results are presented relative to the most detailed DBS electrode model (Model #1). The greatest differences in the DBS voltage distribution were noted with the electric potential source implementation, especially when explicitly modeled inactive contacts were included in the model (Fig 4).

**Fig 5:**
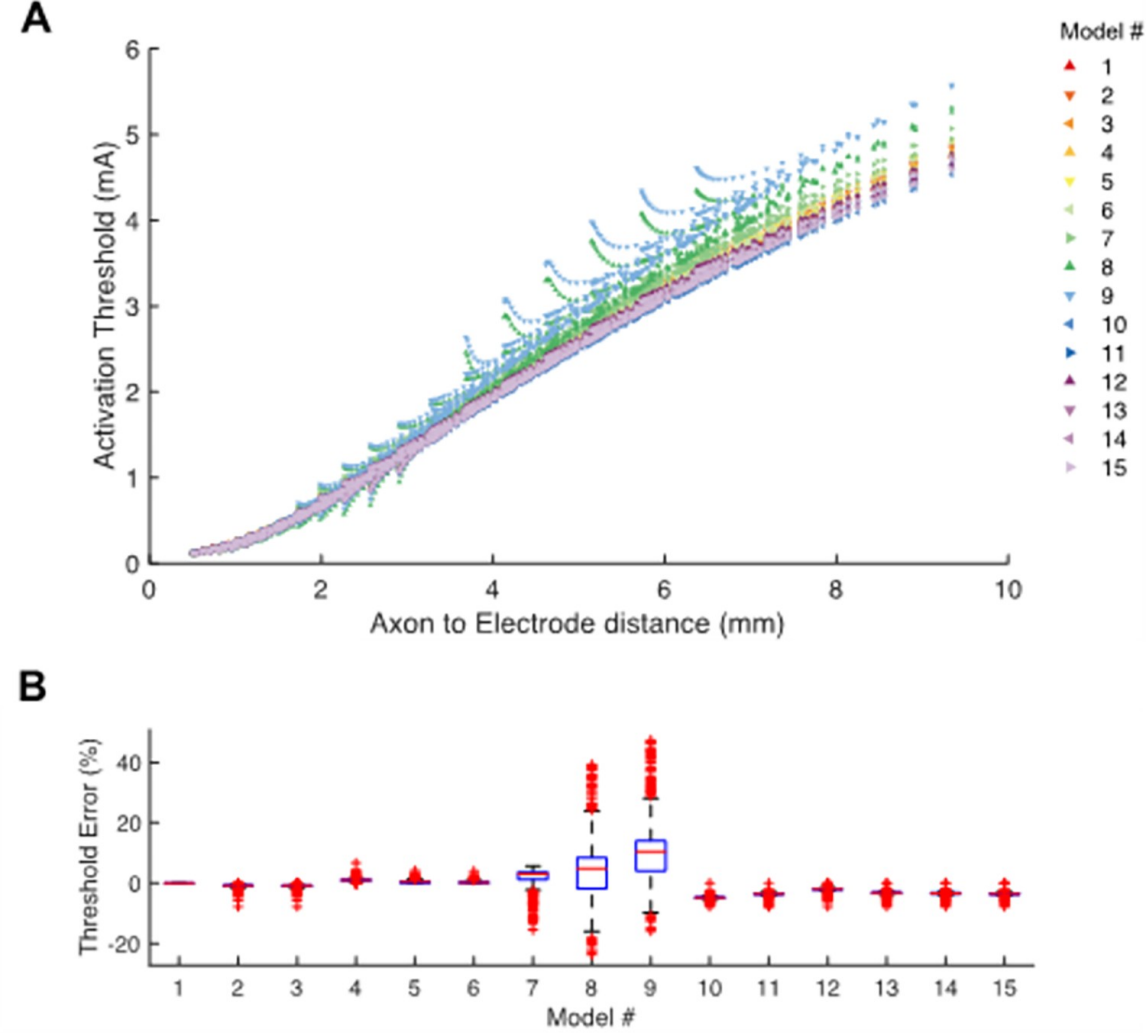
Activation threshold. **A**. Current-distance relationships show the activation thresholds for all axons and all models as a function of distance to the center of the active directional contact. **B**. Activation threshold error shows the relative error in activation threshold of each model compared to Model #1.

Fig 5 shows example current-distance relationships for activation of the axon models. The results are presented for stimulation through a single active directional contact for all the DBS electrode model variants. Activation thresholds increased as the electrode-to-axon distance increased. We defined an activation threshold error as the difference in activation threshold of each model axon, for each DBS electrode model variant, relative to Model #1 (Fig 5B). Differences in activation threshold compared to Model #1, as calculated for individual axons, showed a variability that ranged from -24% to +47%.

**Fig 6:**
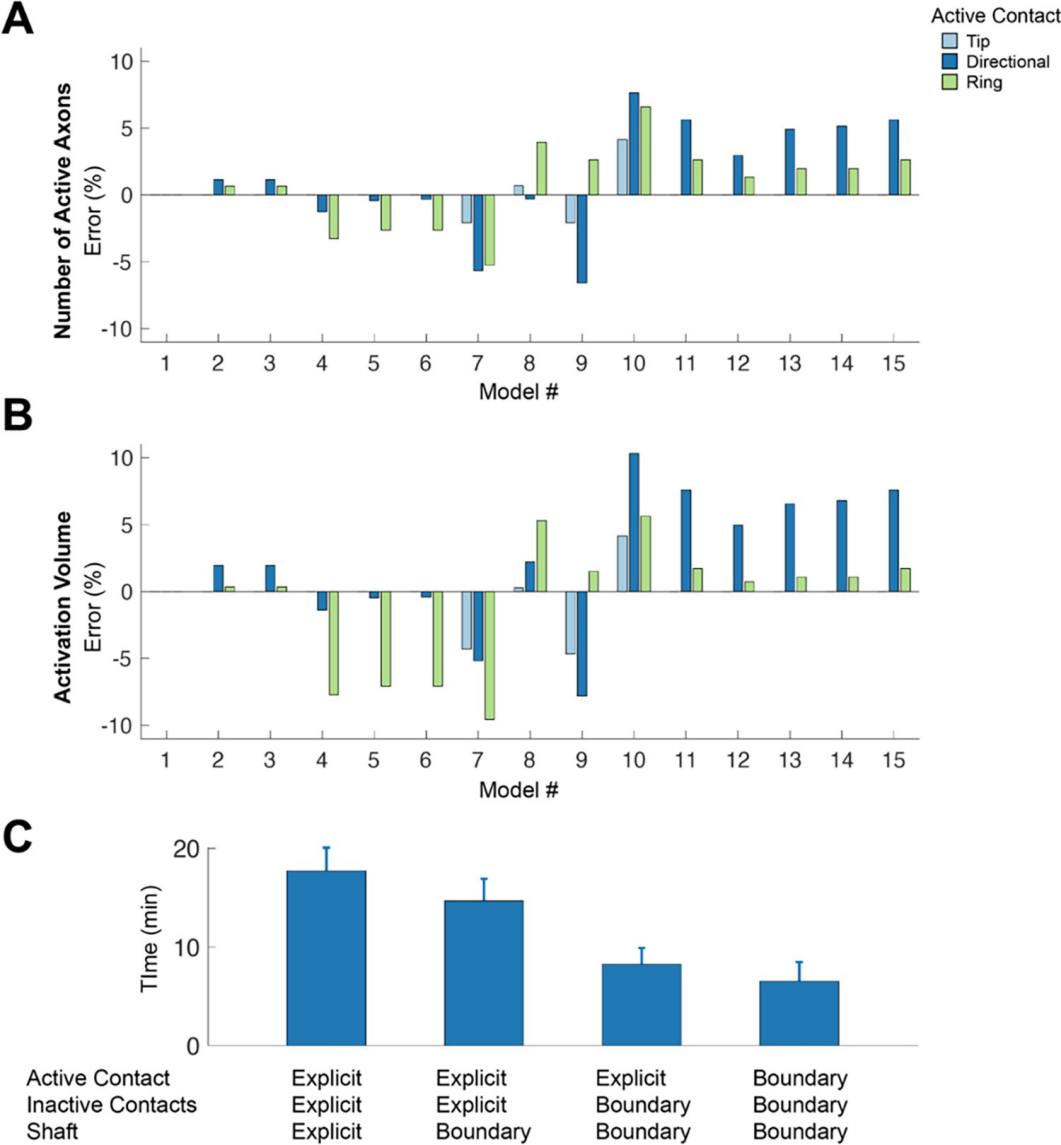
Stimulation spread and computation time. The spread of activation for a 1 mA threshold is calculated as the number of active axons (**A**) and as activation volume (**B**) for the tip contact, the directional contact, and the cylinder contact set as active contact. The relative difference of each model is compared to Model #1. **C**. The average time to build, mesh and solve the model for each level of model geometry option.

A 3D spread of activation was calculated by rotating the axon grid around the DBS lead. Activation was calculated as the total number of active axons, as well as an activation volume. Fig 6 shows example results for a 1 mA 60 μs stimulus delivered through the specified electrode contacts for each of the DBS electrode model variants. The errors presented in Fig 6 are the errors compared to Model #1. In general, the errors increase with decreasing complexity in the model’s geometry. When evaluating a summary metric like the total number of active axons in the population, the errors are reduced to -6.6% to +7.6% (Fig 6A), which is substantially less than the individual axon errors (Fig 5B). The activation volume is a common clinical representation for DBS activation data [12]. The activation volume metric exhibited similar trends in the errors as seen with the total number of active axons, albeit a bit larger error for most of the conditions we examined.

The total computation time to build, mesh, and solve each model variant increased with the number of components on the DBS lead that were explicitly modeled. On average, it took 2.7 times longer to build, mesh, and solve a model with explicit representation of the entire DBS lead compared to a model using boundary conditions for the DBS lead. The computation time for the tip and cylinder contacts is on average 2.1 times longer than a directional contact because of their larger surface areas.

## 4. Discussion

Volume conductor (VC) models of the DBS voltage distribution in the brain are common tools for research on the biophysics of stimulation [9]. In addition, patient-specific DBS models are beginning to be applied in clinical programming [8, 22, 23], as well as the in the generation of hypotheses on which axonal pathways are associated with therapeutic benefit from DBS [13, 24]. However, there remain a wide range of technical questions on the best practices for building and implementing VC models of DBS. Therefore, the goal of this study was to provide a side-by-side comparison of different approaches to DBS electrode model construction (Fig 1), and evaluate the impact of using some different simplifying assumptions in the VC models on predictions of axonal activation from DBS (Fig 3). The results demonstrate substantial differences in the voltage distribution generated by the different DBS VC model variants (Fig 4), which affected individual axonal thresholds (Fig 5). However, the impact of those differences on summary metrics like a generalized activation volume were relatively small (Fig 6). Therefore, the level of model detail that would be appropriate for a specific DBS research project is likely to be dependent on the specific question being addressed. Nonetheless, the results of this study provide information on the technical details and modeling caveats associated with different implementation strategies for simulating clinical DBS electrodes.

The focus of the analyses in this study was on the representation of the DBS electrode contact and the current source. The DBS models we used were intentionally simple, as this level of VC model represents the general platform used in clinical DBS research [13, 23]. We defined the most detailed representation that we implemented as the standard for comparison (Model #1). However, it should be noted that there is no ground truth for modeling the geometry of DBS leads or the implementation of the applied stimulation current. We propose that the point current source is the most realistic source, as it simulates the current distribution through the electrode contact via a simulated weld point connecting the contact to the stimulator. However, none of the model sources are technically correct. All cases are modeled to the steady state solution for time at infinity, and do not account for the heterogeneous distribution of current over time [15]. In addition, the electrode-electrolyte interface is not included in these models [25], so the current density distribution on the contact is not represented correctly. However, all of the models do show an increased current density at the edges and corners of the electrode contact which is a well-established phenomenon [26] (Fig 2).

The DBS voltage distribution generated by the majority of the model variants is comparable to Model #1, with the exception of examples with explicitly modeled inactive contacts (Fig 4). Explicit representation of the inactive contacts introduces substantial variance in the voltage distribution near the contacts, and subsequently translates into discrepancies in the axonal threshold calculations (e.g. Models #8 and #9 in Fig 5A and 5B). However, nearly all of Models #2–15 show some variance in the voltage distribution, which did not always translate into effects on the axonal threshold calculations. This is likely because the neural response to electrical stimulation is not directly associated with the extracellular voltage, or the electric field, but instead the second spatial derivative of the extracellular voltage distribution [27]. So if the variance in the voltage distribution all has either a negative bias or a positive bias, there will be minimal impact on axonal thresholds. However, if there is both positive and negative variance in the voltage distribution, axonal thresholds will exhibit greater differences.

Two general strategies exist for using finite element VC models in clinical DBS research software tools. The most simple approach is to store precompiled solutions of the voltage distribution for each contact of each electrode design, and then access those solutions when needed by the DBS software [24]. However, this approach requires accepting many simplifying assumptions about the tissue medium of the brain and/or the location of the DBS electrode in the brain. Alternatively, the electric field solution can be solved on demand by the DBS software, which requires waiting for the computer to process the simulation, but can enable much more detailed representations of the tissue conductivity and anisotropy [28]. As such, implementation of Model #1 may make more sense when using precompiled solutions, while Model #15 would be a more efficient option when using on demand simulations.

## 5. Conclusion

Volume conductor models of the voltage distribution in the brain generated by DBS electrodes are commonly used in clinical research studies. The goal of this study was to provide a side-by-side comparison of different approaches to modeling the DBS electrode contacts and current sources, and document how those model construction decisions affect estimates of the voltage distribution and axonal activation. The different DBS VC models generated substantial differences in their voltage distributions and axonal thresholds. These results suggest that attention should be paid to the representation of the stimulating contact when constructing a DBS VC model for a specific research application.

## Supporting information

S1 Table Source implementation. (DOCX)
