# Supplementary material for: Comparison of methodologies for modeling directional deep brain stimulation electrodes
Source: PLoS One. 2021 Dec 15;16(12):e0260162. doi: 10.1371/journal.pone.0260162 (PMC8673613; doi:10.1371/journal.pone.0260162)
Supplement: S1 Table — (DOCX) [file pone.0260162.s001.docx]

Supplemental Table 1 – Source Implementation

| **Source** | **Active contact current density** |
| --- | --- |
| Point Current Source | ec.Jx * ec.nx + ec.Jy * ec.ny + ec.Jz * ec.nz |
| Boundary Current Source  Current Density  Floating Potential | (up(ec.Jx) * ec.nx + up(ec.Jy) * ec.ny + up(ec.Jz) * ec.nz) - (down(ec.Jx) * ec.nx +  down(ec.Jy) * ec.ny + down(ec.Jz) * ec.nz) |
| Electric Potential | ec.unx * (down(ec.Jx) - up(ec.Jx)) + ec.uny * (down(ec.Jy) - up(ec.Jy)) +  ec.unz * (down(ec.Jz) - up(ec.Jz)) |
